# Dual-Kinetic Relaxation Spectroscopy for Discrimination of Volatile Organic Mixtures via Finite-Size Surface Gratings

**DOI:** 10.1021/acsomega.6c08719

**Published:** 2026-10-15

**Authors:** Narimane Hassaine, Luca Moretti, Riccardo Castagna, Davide Bizzaro, Oriano Francescangeli, Costanzo Di Perna, Andrea Di Donato, Daniele E. Lucchetta

**Affiliations:** † Università Politecnica delle Marche, Facoltà di Ingegneria, Dip. SIMAU, Optoacoustic Lab, via Brecce Bianche, 12, Ancona 60131, Italy; ‡ Ecole Nationale Supérieure des Technologies Avancées, Department Genie Mecanique Et Productique, Cité Diplomatique Ex Centre Biomédical, Bordj El Kiffan 16012, Algerie; § Istituto di Nanotecnologia Consiglio Nazionale delle Ricerche, NANOTEC, Via Monteroni, Lecce 73100, Italy; ∥ 710799 Consiglio Nazionale delle Ricerche Area di Ricerca di Firenze, Istituto di Scienza del Patrimonio Culturale, via Madonna del Piano 10, Sesto Fiorentino 50019, Italy; ⊥ Consiglio Nazionale delle Ricerche (CNR), URT-DSU@UNICAM, PHOSMAT Lab, via Sant’Agostino, 1, Camerino 62032, Italy; # 9294 Università Politecnica delle Marche, Facoltà di Scienze, Dip. DISVA, Applied and Molecular Genetics Lab, via Brecce Bianche, 12, Ancona 60131, Italy; ∇ Università Politecnica delle Marche, Facoltà di Ingegneria, Dip. SIMAU, Advanced X-ray Diffraction Lab, via Brecce Bianche, 12, Ancona 60131, Italy; ○ Università Politecnica delle Marche, Facoltà di Ingegneria, Dip. DIISM, via Brecce Bianche, 12, Ancona 60131, Italy; ◆ Università Politecnica delle Marche, Facoltà di Ingegneria, Dip. DII, via Brecce Bianche, 12, Ancona 60131, Italy

## Abstract

Discrimination of chemically similar volatile organic compounds (VOCs) in gas mixtures remains a challenge for traditional optical refractive index sensors. In this work, we used a surface holographic grating relaxation technique to monitor the transport kinetics of VOCs. By analyzing the decay of the diffraction efficiency, we extract two independent relaxation time constants. These constants arise from the interplay between the local diffusion of the gas molecules through the material and the geometric aperture losses of the sensing surface (diffusion of molecules in air underneath the grating sensor). These parameters provide a unique kinetic fingerprint for individual species. Mapping these values in a 2D inverse-time state space reveals distinct, nonoverlapping clusters. Furthermore, in the presence of multicomponent gas molecules, by mapping the relaxation lifetimes of pure volatiles and their binary mixtures, we identify a predictable transition in the state space. This approach enables the discrimination between individual volatile compounds and mixtures (e.g., methanol-ethanol) and, in principle, allows their respective concentrations to be quantified and monitored over time with a high degree of sensitivity. This dual-parameter approach uses the finite-size effects of the probe beam, traditionally considered an experimental limitation, to enhance sensor selectivity, offering a robust framework for miniaturizing complex gas-phase analysis.

## Graphical abstract

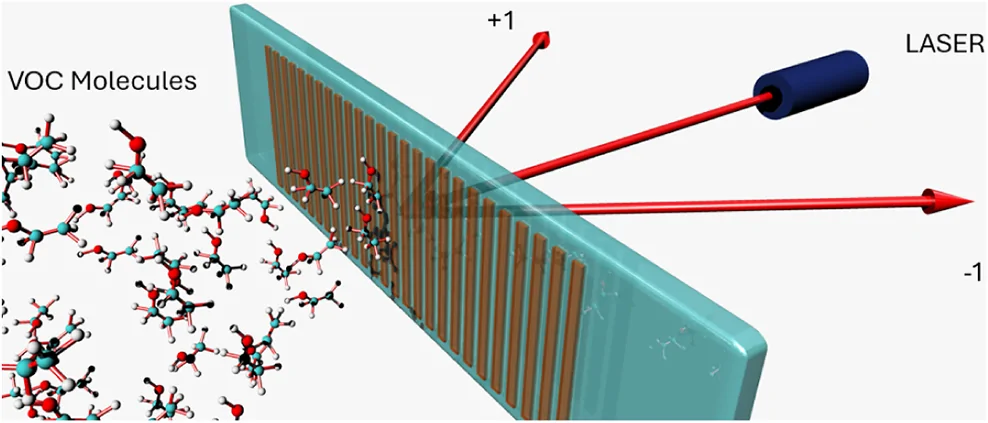

## Introduction

Volatile organic compounds (VOCs) are among the most important classes of atmospheric pollutants due to their adverse effects on human health, industrial safety, and environmental quality. Consequently, the development of highly sensitive, selective, and low-power sensing technologies has become an active research area. In this context, sensors for measuring gas mixtures are of considerable importance. Currently, VOC mixtures are detected using two primary approaches: analytical laboratory separation (which untangles the individual gases in the mixture) and sensor-based direct measurement (which either reads the total VOC concentration or uses data patterns to identify the components).^1^ The first case includes the gas chromatography–mass spectrometry and the proton-transfer-reaction mass spectrometry.^2,3^ On the other hand, the second approach is based on photoionization detectors, flame ionization detectors, and metal oxide semiconductor sensors.^3,4^ The choice of the method depends on whether a highly precise chemical breakdown or real-time, portable monitoring are needed. A great effort has also been done to move toward miniaturized sensors. Different solutions have been developed to identify specific gases within a mixture, on-site, without massive laboratory setup, such as electronic noses, miniaturized gas chromatography, portable photoionization detectors based on nanoporous silica traps and bioinspired sensors.^4–7^ Recent advances have focused on improving both the sensitivity and discrimination capabilities of sensor platforms through innovative material engineering^8^ and sensor-array concepts. An example was described by Fu et al.^9^ which reported a multifunctional VOC sensor based on cascaded quartz crystal resonators coated with different metal–organic films. The authors developed a hybrid sensing platform capable of acquiring multiple sensing parameters from four parallel-connected resonators in a single frequency sweep. A different solution for the selective detection of multiple gases was developed by Nam et al.^10^ using a visible-light-driven chemoresistive gas sensor array based on ISO type-I heterojunction. The heterostructure exploited efficient charge transfer under blue-light illumination to enhance room-temperature gas sensing performance while avoiding the high power consumption typically associated with heated metal-oxide sensors. In this research area, optical sensors represent a rapidly growing technology. Regarding the analysis of mixtures, optical technologies offer unique advantages, alongside important limitations,^5,11,12^ Optical sensors for mixture sensing rely primarily on three distinct architectures. The first is infrared spectroscopy, which leverages the unique ″fingerprint″ absorption spectrum in the mid- or near-infrared range. In the presence of a mixture, nondispersive infrared sensors utilize narrow optical filters to target specific wavelengths. Alternatively, Fourier Transform Infrared (FTIR) spectrometers scan a broad spectrum simultaneously, employing deconvolution software to resolve overlapping numerical peaks from different gases.^13^ The second architecture consists of colorimetric sensor arrays based on chemical dyes or functionalized porous matrices. Here, a mixture generates a unique composite color pattern that can be analyzed to identify the constituent gases.^11,12^ Finally, functionalized integrated optical sensors guide light through a microscopic waveguide coated with a polymer layer. When VOCs are absorbed into the polymer, they alter its refractive index, thereby modifying light wave propagation and its interference.^14–16^ In presence of gas mixtures, optical sensors have some disadvantages. Spectral overlapping can appear when dealing with similar molecules having overlapping infrared absorption lines. In addition, ambient humidity can create giant spectral peaks that can completely mask or distort the tiny optical signals from VOC mixtures. High-precision spectroscopy requires complex setup and in some cases bulky path lengths are required to detect low concentrations of gas mixture.^17–21^ In this context, optical sensors based on holographic diffraction gratings and forced light scattering^22^ offer an attractive alternative to detect VOCs through miniaturized systems. Holographic gratings can operate through direct modulation of the diffracted wavelength, intensity, or angular response induced by changes in the refractive index, thickness, or volume of a functionalized sensing layer. They combine simple optical interrogation, high sensitivity to refractive-index and volume changes, and compatibility with functional polymers, hydrogels, and MOF-based materials.^11^ Recent developments have demonstrated VOC detection through holographic transmission gratings, Bragg gratings, and photopolymer-based holographic structures. They enable low-cost, label-free sensing and, in some cases, discrimination of single VOC species through their characteristic diffusion dynamics within the detection layer.^23^ In particular, for VOC sensing, holographic gratings present several advantages. First, the periodic nanostructure amplifies the small physicochemical changes occurring within the sensing material, resulting in high sensitivity to analyte adsorption. Second, functional polymers, hydrogels, or MOF-based coatings can be integrated within the grating structure, allowing tailored selectivity toward specific VOC classes.^24^ Third, the spectral response can be monitored without electrical contacts or power-intensive heating elements, facilitating portable and wearable implementations even on microfluidics platforms.^25^ Finally, multiplexed holographic architectures could simultaneously provide wavelength-selective and intensity-based information, opening opportunities for VOC identification through optical fingerprinting strategies analogous to electronic ″noses″, but with substantially simplified instrumentation. Here, we demonstrate the discrimination of volatile organic compounds (VOCs) in gas mixtures, using a surface holographic grating coupled with dual-kinetic relaxation spectroscopy. By analyzing the temporal decay of the diffraction efficiency under specific excitation conditions, we extract two independent, average relaxation time constants. These constants correspond to the local diffusion of gas molecules through the material and their local diffusion in air through the geometric aperture of the sensing surface. Although these parameters provide a unique kinetic fingerprint for individual species, they also enable the discrimination of specific components within VOC mixtures. Mapping these values into a 2D inverse-time state space reveals distinct, nonoverlapping clusters.

## Experiments

The core element of the experimental setup is a tailored holographic thin grating. To record high-quality diffraction gratings, we use a high-speed commercial UV-sensitive photopolymer provided by Anycubic, along with a high power continuous-wave (CW) laser operating in the UV range. A double layer structure is used to prevent the detachment of the grating structure from the supporting glass. The photopolymer is spin-coated onto a standard microscope glass slide to form a thin film approximately 100 μm thick. This film is exposed for 5 min to a uniform UV light and completely photocured. On top of this layer, we deposit another thin substrate in which a one-dimensional interference pattern generated by a Genesis CX SLM UV laser (power = 100 mW, wavelength λ = 355 nm) is recorded. The exposure time is 5 s. The writing setup recall a Mach–Zehnder configuration, where each of the two interfering beams forms a 10° angle with respect to the normal to the glass surface.^23^ After exposure, the residual unpolymerized resin is completely removed by immersing the glass slide in an isopropyl alcohol ultrasonic bath for 5 min. The resulting structure is a well-defined mixed Bragg/Raman-Nath sinusoidal grating, characterized by a strong dielectric contrast between the polymer walls and the surrounding air. The interplay between the Bragg and Raman-Nath regimes enables sufficient light to be directed into the +1 and −1 diffraction orders without significant losses to the lower orders. A low-power He–Ne laser is used as a probe to characterize the diffraction properties of the grating both during and after the recording process.

Figure 1 shows a scheme of the detection system. A representative AFM image of a recorded transmission grating is shown in the inset of Figure 1. The grating features a pitch Λ = 0, 9–1, 0 μ*m*, a depth *h* in the 100 nm range and a diameter of 6 mm. The characterization of the gratings is made by using the atomic force microscope NT-MDT model SMENA-B (provided by Pra.Ma.), equipped with a microscope. The topography is measured in semicontact mode using a silicon tip with a radius of curvature of about 20 *nm*.

**Figure 1:**
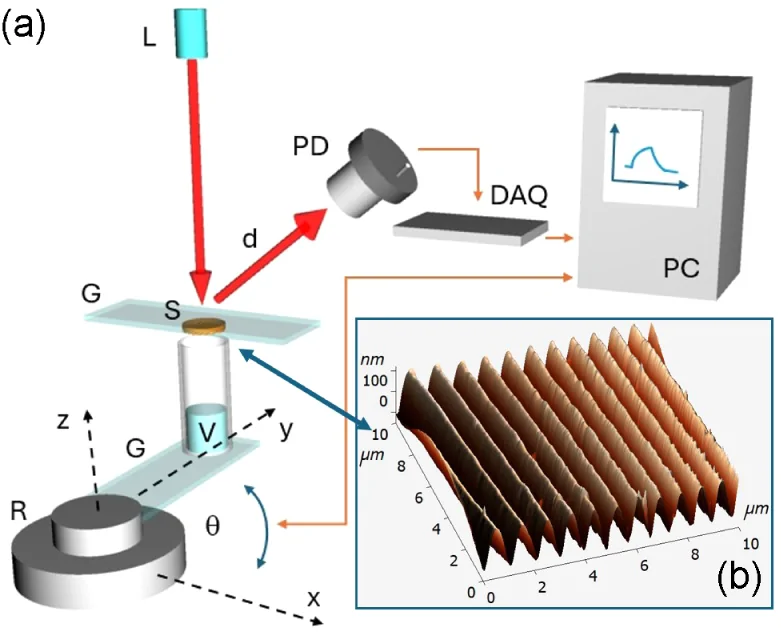
(a) Detection setup: L: laser; PD: photodetector; V: VOC inside the vessel; d: diffracted signal; R: rotational stage; S: grating; DAQ: data acquisition board; PC: personal computer. (b) AFM 3D topography of a typical 1D recorded structure.

In Figure 1 a low-cost CW laser source operating at 632 *nm* is directed perpendicularly onto the central region of the grating, forming a 1 *mm* diameter spot. This configuration produces four first order diffracted beams (two transmitted, two reflected). One of the first-order reflected beams is monitored in real time by a photodiode (PD1), connected to a 16-bit NI acquisition board.

A fast computer-controlled rotation stage, rotating by an angle of 90° around the *z*-axis, was used to position a cylindrical glass vessel directly below the grating. The vessel had an internal diameter of 10 mm, a height of 24 mm, and an upper opening of 6 mm. It was filled with 20 μL of the selected VOC liquid precursor.

After filling the vessel, the acquisition system waits 10 s before starting the entire exposure sequence, in order to reach a steady state condition for the vapor concentration at the vessel aperture. This allows us to experimentally implement a pulse excitation as defined in the model section (see Figure 2a). The vessel is then brought under the grating by a fast motor rotation, with the top of the vessel positioned at a distance *h* = 1 mm from the grating itself. Because the mechanical displacement is fast compared with the characteristic response time of the grating–VOC system, the molecular input incident on the grating can be approximated as a step-like excitation.

**Figure 2:**
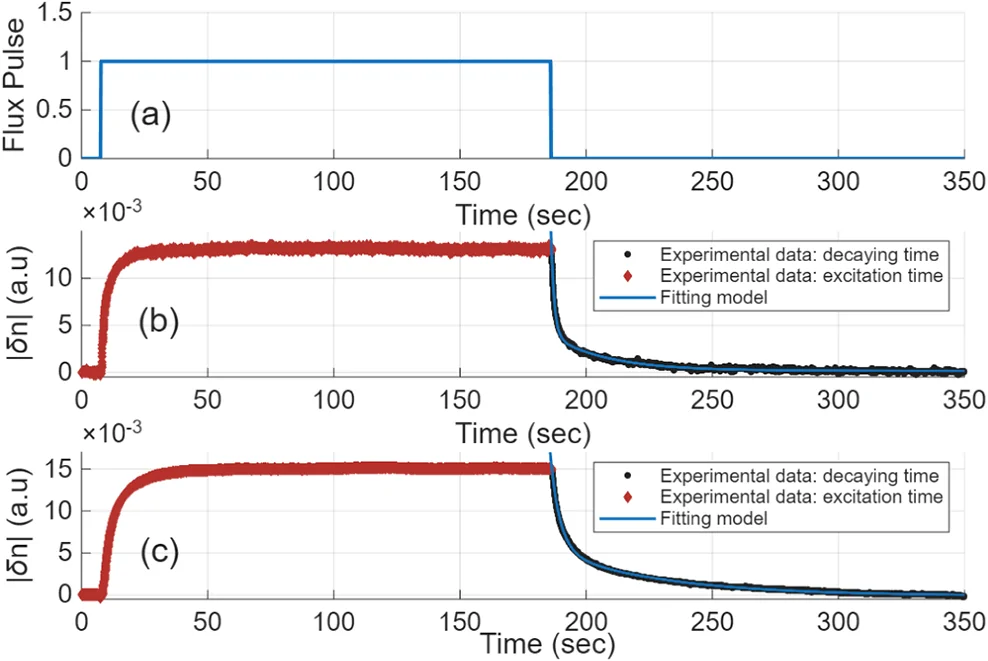
Flux of gas molecules incident on the grating is approximated by a step function in time (a). Below are reported the relative refractive index changes for methanol (b) and ethanol (c), respectively. The response to the pulse excitation is highlighted in red. The black points describe the relaxation response of the system, after removal of the vessel, which is fit with a biexponential model.

Each run consists of a 210 s absorption phase, during which the optically interrogated region of the grating is exposed to the VOC vapor plume, followed by a 180 s recovery phase, during which the grating is moved away from the detection position. During this recovery phase the liquid precursor continues to evaporate from the vessel; therefore, the VOC source itself is not switched off. Rather, the VOC flux incident on the probed region of the grating is effectively interrupted because the grating no longer intercepts the vapor. A single run lastes 390 s, i.e., approximately 7 min including initial stabilization and handling time. The run is repeated several times until complete evaporation of the liquid precursor, and each run is stored as raw data on a PC for subsequent analysis.

Humidity and temperature are continuously monitored during each experiment. The average values are approximately 42% relative humidity and 24 °C, with no significant variations observed throughout the experimental sessions.

## Relaxation Spectroscopy Model

Relaxation behavior can be modeled by introducing a simplified kinetic model involving two different populations: the number of molecules per unit of volume *N*_*p*_ diffused inside the grating and the number of molecules in the air region surrounding the polymer interface *C*_*a*_.^23^ Different models have been proposed to describe the kinetic behavior of gas molecules during diffusion and adsorption in solids.^26–28^ These models differ both in their mathematical structure and in their physical interpretation. They are commonly classified into four main categories: adsorption–reaction kinetics models, empirical models, diffusion-based models, and models describing adsorption on active sites.^27^

Here we assume that the interaction between the gas molecules and the polymer involves only the physical diffusion of molecules without any chemical reaction. This assumption is supported by the reversibility of the process observed during the experiments. In addition, under the condition of relatively low ambient concentrations, the temporal evolution of the molecular density *N*_*p*_ interacting with the grating can be represented by a diffusion-based scheme.^26–28^ This approach relies on two key assumptions: (i) at equilibrium, the number of adsorbed molecules is proportional to the ambient concentration of the analyte, and (ii) the adsorption rate is proportional to the difference between the equilibrium number of adsorbed molecules and the number currently present on the surface.

According to the configuration of the experimental detection setup (see Figure 1), we describe the physical interaction between gas molecules diffusing in the air and through the polymer, using three coupled rate equations. These equations describe the dynamic of two populations: the molecules within the chamber *C*_*a*_(*t*) and the molecules diffused inside the polymer *N*_*p*_(*t*):

1 $$\frac{dC_{a}\left(t\right)}{dt}=J_{S}\left(t\right)-\frac{dN_{p}\left(t\right)}{dt}-\frac{dC_{ext}\left(t\right)}{dt}$$

2 $$\frac{dN_{p}\left(t\right)}{dt}=\frac{1}{\tau_{p}}\left(\gamma C_{a}\left(t\right)-N_{p}\left(t\right)\right)$$

3 $$\frac{dC_{ext}\left(t\right)}{dt}=\frac{1}{\tau_{a}}\left(C_{a}\left(t\right)-C_{air}\left(t\right)\right)$$

The term *J*_*S*_(*t*) is the flux of molecules that evaporate from the surface S of the liquid, assumed to be uniform on the grating surface and with a temporal evolution depending on the excitation mechanism. The term τ_*p*_ is the diffusion time constant of the gas molecules through the polymer. The term $N_{p}^{eq}=\gamma C_{a}\left(t\right)$ represents the fraction of molecules that diffuse through the grating at equilibrium, depending on the ambient concentration *C*_*a*_. For relatively small ambient concentrations and when the external concentration is almost a step-function of constant-value, eq 3 leads to a ″pseudo first-order″ model.^26^ The term *dC*_*ext*_(*t*)/*dt* is a loss rate due to the molecules diffusing out of the chamber through the apertures of the sensing chamber, with τ_*a*_ being the loss time constant. The sensing region, illuminated by a collimated laser beam, is limited to a small area compared to the excitation surface (see Figure 1). The loss rate is assumed to be proportional to the difference between the gas concentration inside the chamber *C*_*a*_ and the gas concentration outside the chamber *C*_*air*_. The latter is approximated to a zero concentration. The system of coupled equations can be rewritten in a more compact matrix notation, as follows:

4 $$\left(\begin{array}{c} \overset{˙}{C_{a}\left(t\right)}\\ \overset{˙}{N_{p}\left(t\right)} \end{array}\right)=A\left(\begin{array}{c} C_{a}\left(t\right)\\ N_{p}\left(t\right) \end{array}\right)+\left(\begin{array}{c} J_{S}\left(t\right)\\ 0 \end{array}\right)\;\mathrm{where}$$

5 $$A=\left(\begin{array}{c} -\frac{\gamma }{\tau_{p}}-\frac{1}{\tau_{a}}\frac{1}{\tau_{p}}\\ \frac{\gamma }{\tau_{p}}-\frac{1}{\tau_{p}} \end{array}\right)$$

The matrix **A** describes the state of the system and its eigenvalues λ_1_ and λ_2_ characterize the dynamic response of the system. They depend on the diffusion time constant τ_*p*_, the loss rate τ_*a*_ and the fraction of gas molecules γ that diffuse within the polymer. They represent a unique signature of the molecular species interacting with the polymeric grating. Therefore, their values can be used to differentiate individual gases. In addition, they can also be applied to analyze gas mixtures. In this case, the time constants assume an “average” value that depends on the relative concentration of the two substances under consideration. During the gas exposure time, the air surrounding the grating will slightly change its refractive index due to the presence of an external gas concentration *C*_*a*_(*t*) at the air–polymer interface. At the same time, the refractive index of the polymer at the interface with air will vary a little due to the diffusing molecules through the surface grating. According to the Clausius-Mossotti equation^29^ and under the assumption of low concentrations, the relative variation of the refractive index δ*n* can be written as a linear combination of concentrations *C*_*a*_ and *N*_*p*_, i.e., δ*n*(*t*) = α_1_ *N*_*p*_(*t*) – α_2_ *C*_*a*_(*t*). The terms α_1_ and α_2_ are functions of the polarizability and refractive indices of gas and air, respectively. During relaxation time, the flux of molecules *J*_*S*_ in the coupled eq 5 is zero, and its solutions can be described as a linear combination of exponential functions, having inverse time constants λ_1_ and λ_2_. Due to the geometry of the grating and the working wavelength (see Figure 1), it is possible to approximate the structure as a thin sinusoidal phase reflectance grating,^22^ whose first-order diffraction efficiency can be accurately described by the following equation:^22^

6 $$\eta ={|J_{1}\left(\phi \right)|}^{2},\mathrm{in which},\phi =\frac{2\pi \delta n\left(2h\right)}{\lambda }$$

where δ*n* is the refractive index gap between the air and the polymer, *h* is the depth of the grating, and λ is the laser wavelength in the polymer. The relation between diffraction efficiency and the change in refractive index can be linearized under the assumption of low gas concentrations and by properly choosing the initial working point ϕ. In fact, depending on the wavelength λ, the grating depth *h* and the expected average refractive index gap (δ*n*), the initial value of the phase ϕ could be located at different positions along the nonlinear curve of the diffraction efficiency: the rising edge (A), the peak (B), or the falling edge (C) (see Figure 3). In our system, the initial phase was shifted at the edge (C) of the diffraction response, characterized by higher sensitivity and linearity, by controlling the value of the grating depth. The holographic grating was designed to have a depth of *h* = 100 *nm*. Since very slight variations in its value were present on the surface of the polymerized spot, it was possible to test different locations and find the optimal value of *h* able of providing a better linear response for different gases and concentrations. Furthermore, during the full operation range (from excitation to equilibrium condition when the grating is exposed to VOC) the value of δ*n* was always maintained within the linear region (see Figure 3). In this way, the efficiency η could be fairly approximated to a linear dependence with respect to ϕ, obtaining the following proportionality:

**Figure 3:**
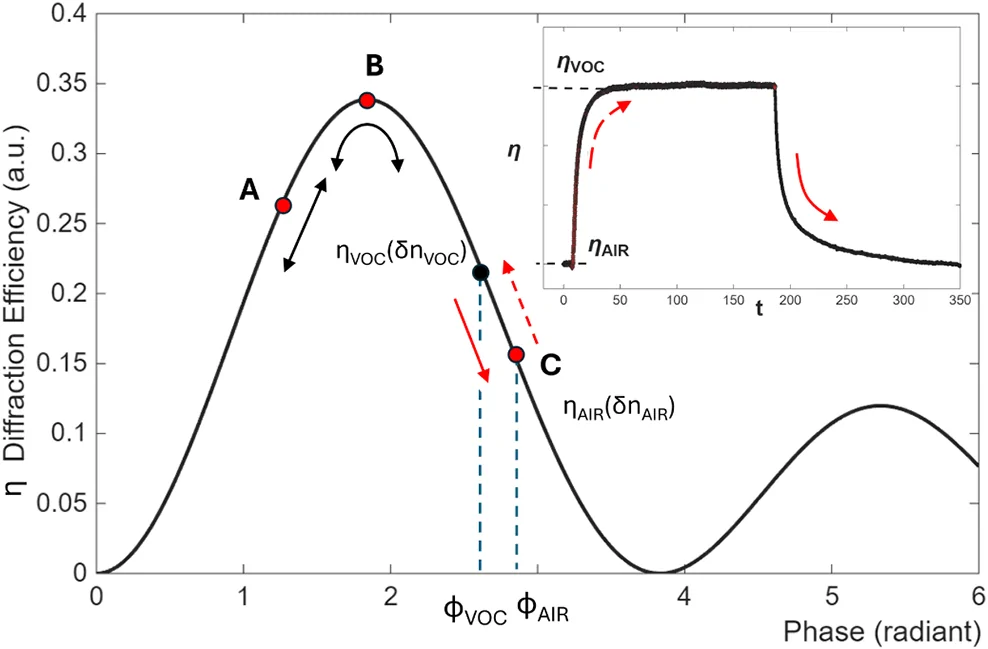
Diffraction efficiency response for a thin phase grating in which different working points are highlighted along the nonlinear curve: the rising edge (A), the peak (B), or the falling edge (C). Inset: example of time response of the refractive index change during the excitation and relaxation phases assuming a starting working point at the falling edge (C). δ*n_air_* and δ*n_voc_* are the refractive index gap between the surrounding air and the polymer before and after the interaction of the grating with gas molecules, respectively .

7 $$\eta \propto \phi \propto \left(\frac{2\pi \delta n\left(2h\right)}{\lambda }\right)$$

In this work, we tested two gases, methanol and ethanol and a 1:1 mixture of both. The two gases induce a similar refractive index change δ*n* which we are able to discern. From the reference and diffracted intensities, we can derive the diffraction efficiency η in reflection. Once the working wavelength and the geometry of the grating are known, by numerically solving eq 7, the temporal variation of δ*n* is computed.

## Results and Discussion

A typical experimental result concerning diffraction efficiency as a function of time is reported in Figure 4 for methanol. Each curve shows an increase in diffraction efficiency during the absorption phase, followed by a decrease during the recovery phase after the grating is moved away from the ethanol vapor plume. The return of the signal to approximately its initial baseline indicates that each subsequent cycle begins with the grating in a nearly fully recovered state.

**Figure 4:**
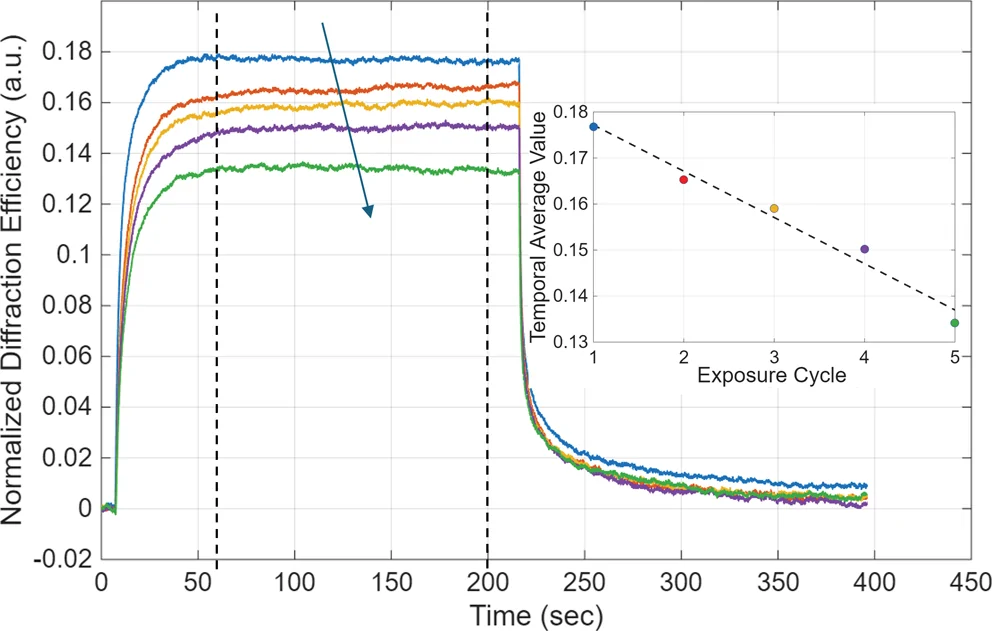
Diffraction efficiency η as a function of time during repeated methanol exposure cycles (temporal sequence indicated by the arrow). The inset shows the linear dependence of the average diffraction efficiency on the number of exposure cycles. The points are calculated as the average of the curve intensity between 60 and 200 s (the two dashed lines), where the system response reached the equilibrium state.

During each absorption phase, the grating is exposed for 210 s to an approximately constant effective flux of VOC molecules. We assume that the effective local VOC concentration at the grating remains constant during each individual absorption phase. As an example, for ethanol at 24 °C, complete evaporation of 20 μL in ≈56 min corresponds to an average evaporation rate of about 1.0 × 10^–7^ mol s^–1^. This is equivalent to roughly 6 × 10^16^ ethanol molecules per second emitted by the vessel. The source flux continues during both the 210 s absorption phase and the 180 s recovery phase; however, during the recovery phase the grating is moved away from the detection position and therefore no longer intercepts the vapor plume. Consequently, the molecular input incident on the grating can be approximated as a rectangular pulse, remaining on during the 210 s absorption phase and effectively off during the subsequent 180 s recovery phase. However, the optical readout probes only a circular region of the grating with a diameter of 1 mm, corresponding to an area of about 0.785 mm^2^. Therefore, the relevant quantity for the optical measurement is not the total molecular flux emitted by the vessel, but the effective local molecular flux intercepted by the optically probed region when the grating is in the detection position. For a vessel aperture of 6 mm in diameter, a vessel–grating distance of 1 mm, and mild ambient ventilation, the effective ethanol flux incident on the 1 mm-diameter optical spot is estimated to be of the order of Φ_grating_ ≈ 10^15^ molecules per second. During the 210 s absorption interval, this is equivalent to approximately *N*_cycle_ ≈ 10^17^ molecules of ethanol incident on the optically interrogated surface of the grating (or equivalently to a concentration *C*_eff_ ≈ 7.0 × 10^3^ ppm). During the recovery phase, the grating signal returns close to its initial baseline before the next exposure. Therefore, each run can be treated as a new absorption event. At the same time, the liquid precursor continues to evaporate from the vessel, so that successive runs are performed on a progressively depleted liquid reservoir. For ethanol, however, this depletion does not imply a proportional decrease of the vapor flux at the grating. As long as liquid ethanol remains present, the vapor pressure at the liquid surface is fixed by temperature, while the open-vessel plume is mainly determined by the vapor pressure, the vessel geometry, and the ambient ventilation.

Under the present conditions, the average ethanol volume loss is about 0.36 μL min^–1^. Since one run lasts 390 s, approximately 2.3 μL of ethanol are lost between the beginning of two consecutive runs. This progressive depletion produces only a weak change in the effective exposure conditions: from the first to the last run, the estimated local ethanol concentration at the grating surface decreases from about 7.0 × 10^3^ ppm to approximately 6.8–6.9 × 10^3^ ppm, corresponding to an estimated variation of about 1–2 × 10^2^ ppm. This value should be regarded as the theoretical sensitivity of our detection system. It is important to underline how this claimed theoretical value of sensitivity needs to be confirmed experimentally by a proper calibration made by using a professional FTIR equipment or a commercial PID.

The run-to-run evolution of the fitted kinetic parameters can therefore be compared with a small but quantifiable decrease of the effective VOC exposure. In order to fully characterize the response of the system, according to the previous considerations, the time-dependent molecular flux incident on the grating can be well approximated by a step function in time, since movements of the glass vessel can be considered as instantaneous with respect to the time constant involved in the evolution of the system (grating + VOC). Under this condition, eq 3 can be conveniently solved in the Laplace domain,^30^ providing the following solutions in the time domain for the relative refractive index change δ*n*(*t*):

8 $$\delta n\left(t\right)=\delta n_{1}\cdot e^{-\lambda_{1}\cdot t}+\delta n_{2}\cdot e^{-\lambda_{2}\cdot t}$$

As highlighted in the inset of Figure 3 and in Figure 2 (refractive index evolution) and described by eq 8, a typical temporal response of the system is characterized by a relatively fast transient decay followed by a slower decay. Focusing on the desorption phase and, therefore, the relaxation dynamics, the refractive index behavior is described by the inverse time constants λ_1_ and λ_2_, which are the eigenvalues of the matrix **A** describing the state of the dynamic system, with λ_2_ ≪ λ_1_ (see eq 5). The latter condition leads to the following expressions:

9 $$\delta n\left(t\right)={\delta n}^{eq}\cdot \left(\frac{\lambda_{2}}{\lambda_{2}-\lambda_{1}}\cdot e^{-\lambda_{1}\cdot t}+\frac{-\lambda_{1}}{\lambda_{2}-\lambda_{1}}\cdot e^{-\lambda_{2}\cdot t}\right)$$

in which δ*n* ^*eq*^ is the relative change in the refractive index at equilibrium. We can study the evolution of the molecule desorption from the grating surface, considering the values of the two inverse time constants with λ_2_ related to the diffusion of the mixture molecules from the air close to the grating and λ_1_ related to the desorption of the molecules diffused through the polymer. At very low gas concentrations and thus for γ ≪ 1, the eigenvalues of matrix **A** are independent of gas concentration and tend to 1/τ_*a*_ and 1/τ_*p*_, respectively. In general, their values depend on the concentration of gas through the fraction of molecules that diffuse into the grating γ.

In Figure 5 we report a plot of λ_1_ vs λ_2_ for the analyzed VOCs: methanol, ethanol and different mixture ratios. Each point corresponds to a complete 6.5 min acquisition run, as described in the experimental section. The data relative to mixtures ratios are confined between the pure ethanol and methanol clusters. The behavior is clearly nonlinear due to the different physical parameters characterizing the two compounds. This distribution is indeed related to their vapor tension and molecules dimensions. Since methanol is characterized by a higher vapor tension and smaller molecules, its inverse time constants are higher in value. After each run, the general behavior of the binary mixture shows a convergence toward ethanol as underlined by the arrows in Figure 5.

**Figure 5:**
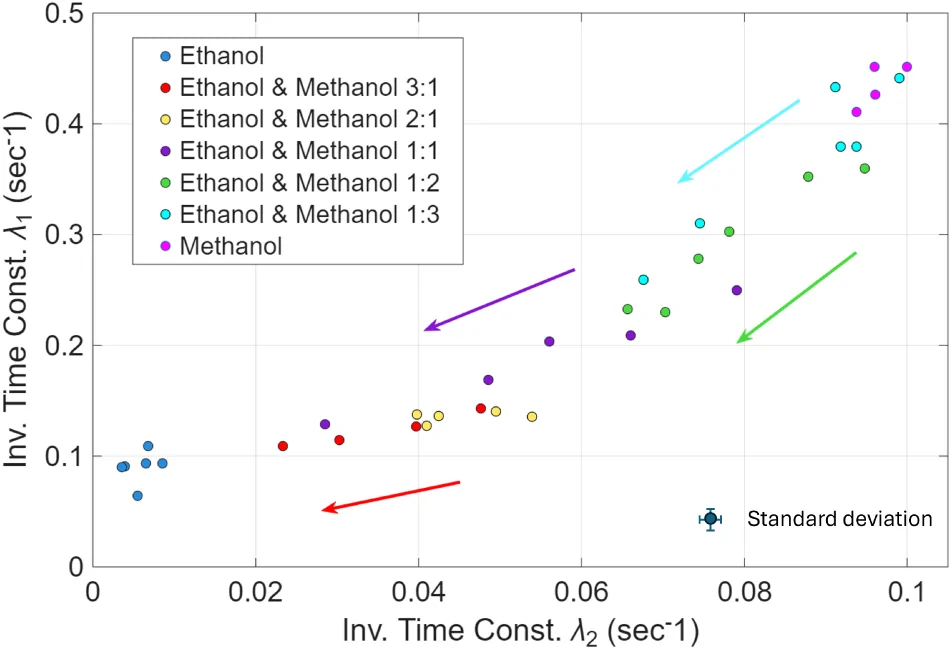
Plot of parameter λ_1_ versus λ_2_ for methanol (blue dots), ethanol (red dots), and their binary mixture (green dots). Each data point represents a different run and different value of concentration. After each run, the general behavior of the binary mixture shows a convergence toward ethanol as underlined by the colored arrows. The standard error (SE) was evaluated for each experimental run. The resulting values are SE = ±0.026 *s* ^–1^ for λ_1_ and SE = ±0.0010 *s* ^–1^ for λ_2_.

The distinct regions in the 2D plot allow us to differentiate between individual VOCs and their mixtures as well. This result supports the assumption introduced in this work. Under the current operating conditions and measured range of concentrations, we treated the mixture as a single, equivalent gas characterized by an “effective” relaxation constants, while keeping a simplified first-order diffusion model. In this way, the time constants for the mixture (λ_1_ and λ_2_) represent ″effective″ values that implicitly takes into account the competitive adsorption effects in the mixture at the polymer surface. Their values depend on the relative gas concentration in the mixture. Throughout the total experimental time, the points relative to each mixture appear to be quite stable and confined within their corresponding regions with a dispersion for λ_2_ slightly higher than λ_1_.

## Conclusions

In this work, we demonstrated a novel dual-kinetic relaxation spectroscopy technique using finite-size surface holographic gratings for the discrimination of chemically similar volatile organic compounds (VOCs) and their binary mixtures. By modeling the molecular transport dynamics with a coupled two-population rate equation system, we showed that the temporal decay of the reflection diffraction efficiency provides a unique kinetic fingerprint for individual gas species and for mixtures of two gases. From this approach, we can extract two independent average relaxation time constants that govern the dynamics of the system. These eigenvalues reflect the physical interplay between the local diffusion of VOC molecules through the grating and the losses through the geometric apertures. When mapped onto a 2D inverse-time state space, these parameters yielded distinct, nonoverlapping clusters for individual gases and mixtures. This methodology transforms the finite-size effects of the probing beam (traditionally treated as an experimental limitation) into a parameter that significantly enhances sensor selectivity. By operating by optical interrogation of the diffraction efficiency without requiring power-intensive heating elements or complex, bulky spectroscopic setups, this platform offers a potential highly sensitive, low-cost alternative for gas-phase analysis. Future work will focus on scaling this dual-parameter approach to complex multicomponent mixtures that contain more gas species, as well as expanding the platform into automated microfluidic sensing arrays for real-time environmental monitoring.

## Notes

The authors thank Prof. Simona Sabbatini and Mr. Matteo Foria for the valuable scientific discussions.
